# Fibrinogen Longmont: A Clinically Heterogeneous Dysfibrinogenemia with Discrepant Fibrinogen Results Influenced by Clot Detection Method and Reagent

**DOI:** 10.1055/s-0041-1740644

**Published:** 2022-01-24

**Authors:** Becky Leung, Joanne Beggs, Jane Mason

**Affiliations:** 1 Department of Haematology, Pathology Queensland, Royal Brisbane and Women's Hospital, Brisbane, Australia; 2 School of Medicine, Griffith University, Gold Coast, Australia; 3 Queensland Hemophilia Centre, Royal Brisbane and Women's Hospital, Brisbane, Australia; 4 Faculty of Medicine, University of Queensland, Brisbane, Australia

Fibrinogen is a 340 kDa glycoprotein composed of three pairs of polypeptide chains. Thrombin cleaves the N-terminal Aα and Bβ chains, exposing binding sites that enable polymerization and cross-linking to form a fibrin clot. Fibrinogen disorders can be quantitative or qualitative, inherited or acquired. Current International Society on Thrombosis and Haemostasis guidelines recommend a stepwise approach to diagnosis of congenital fibrinogen disorders, involving clot-based activated partial thromboplastin time (APTT) and prothrombin time (PT), and fibrinogen measurement by functional and antigenic assays. 1 Congenital dysfibrinogenemias encompass over 400 abnormal variants, typified by abnormal functional assays with discordant normal antigenic levels. They are clinically diverse and can be asymptomatic or associated with bleeding and thrombotic sequelae. 1 2 3

We report two adult siblings ( Table 1 ) diagnosed with the fibrinogen Longmont variant after an unmeasurable Clauss fibrinogen (FibC) was found. Informed consent for publication of this case report was obtained. The 49-year-old female proband had coagulation testing for evaluation of local bleeding following failed insertion of a brachytherapy rod to treat stage 2B cervical cancer. She was on weekly cisplatin treatment and had no prior history of thrombosis or bleeding. APTT and PT were within reference range on the optical endpoint ACL TOP (Instrumentation Laboratory, Massachusetts). PT-derived fibrinogen (FibD) was mildly reduced; however, FibC was unmeasurable using bovine HemosIL QFA Thrombin on the ACL TOP. Her FibD had previously been normal and this was the first FibC performed. FibC is the most accurate clot-based assay to determine functional levels of fibrinogen, compared to FibD, which is an indirect measurement. 1 The thrombin clotting time (TCT) was mildly prolonged with a normal Reptilase time. Full blood count revealed moderate thrombocytopenia (platelet count 87 × 10 ^9^ /L) and anemia (hemoglobin 80 g/dL), with an unremarkable blood film. Liver function testing and serum protein electrophoresis were normal.

**Table 1:** Coagulation assays of proband and brother at diagnosis

Test	Method and reagent	Proband	Brother	Reference range
PT	ACL TOP Werfen Recombiplastin 2G	13	9	9–13 s
APTT	ACL TOP HemosIL SynthASil	26	27	24–39 s
TCT	ACL TOP HemosIL QFA Thrombin	22	22	11–17 s
Reptilase time	ACL TOP Diagnostica Stago Reptilase	19	Not done	<20 s
FibD	ACL TOP Werfen Recombiplastin 2G	1.5	1.3	1.7–4.5 g/L
FibC	ACL TOP HemosIL QFA Thrombin	<0.4	0.5	2.0–4.5 g/L
FibC	ACL TOP STA-Thrombin	5.5	Not done	2.0–4.5 g/L
FibC	STAR Analyzer STA-Thrombin	5.9	4.3	2.0–4.5 g/L
Fibrinogen immunologic	Siemens BNII Nephelometer	6.2	Not done	1.8–3.5 g/L

Abbreviations: APTT, activated partial thromboplastin time; FibC, Clauss fibrinogen; FibD, PT-derived fibrinogen; PT, prothrombin time; TCT, thrombin clotting time.

The initial provisional diagnosis was an evolving malignancy-associated disseminated intravascular coagulation. However, her clinical condition did not deteriorate, fibrin degradation products were only mildly elevated, APTT and PT were normal, and platelet count was stable. Cryoprecipitate was given as fibrinogen replacement yielding an unpredictable fluctuating pattern of intermittently normal or high FibC, after relatively modest doses of cryoprecipitate.

In contrast to the functional FibC assay, the antigenic immunologic fibrinogen was normal prior to cryoprecipitate replacement. An acquired paraneoplastic dysfibrinogenemia was subsequently considered. Serendipitously, her brother attended the hospital for an unrelated clinic appointment and was found to have an unmeasurable FibC with a similar coagulation profile to his sister ( Table 1 ). The brother was asymptomatic with no significant bleeding or thrombosis history. The laboratory scientific staff recognized the unique surname and it was established that they were related, providing strong weight for a congenital dysfibrinogenemia.

The FibC for both siblings was normal on the mechanical endpoint STAR Analyzer (Diagnostics Stago, France) using human STA-Thrombin. Subsequently, our laboratory validated the use of STA-Thrombin on the ACL TOP and repeated the female patient's FibC, obtaining a normal result. Examination of ammonium sulfate purified fibrinogen by online reverse-phase electrospray time-of-flight mass spectrometry for both patients, suggested heterozygosity for an Arg- > Cys (-53Da) polymorphism in the Bβ chain. DNA sequencing of exon 4 of *FGB* confirmed that both were heterozygous for fibrinogen Longmont with a point mutation resulting in Bβ166Arg- > Cys ( *FGB* NM_0005141.4:c.586C > T, p.Arg196Cys).

Fibrinogen Longmont was originally described by Lefkowitz et al, 4 with the unusual finding of discrepant results between mechanical and spectrophotometric clot assays. In this original paper, a 37-year-old female proband and her mother had a mild–moderate bleeding phenotype. Testing revealed a markedly prolonged PT, TCT, and Reptilase time on the spectrophotometric ACL3000 + , but normal results on the electromechanical ST4 Analyzer. FibC was normal on the ST4 but was not performed on the ACL3000 + . Sequence analysis of the proband and her mother identified Bβ166Arg- > Cys. 5 Their interpretation was that fibrinogen Longmont was clottable, but the clot was translucent. 4 Subsequent protein analysis indicated that this mutation alters fibrinogen polymerization by disrupting interactions critical for normal lateral association of protofibrils. 6

Grimley et al 7 described a family with fibrinogen Longmont with no bleeding phenotype, and thrombosis in one family member. PT and fibrinogen assay were both unclottable on the optical Sysmex CA7000, and normal on the electromechanical KC10, again suggesting a translucent fibrinogen.

Given the original description of fibrinogen Longmont forming a translucent clot, our ability to obtain a normal FibC result on an optical analyzer with a human thrombin reagent is puzzling. The cleavage target on the fibrinogen molecule for both human and bovine thrombin is identical, as was the concentration of human and bovine thrombin reagents used. It is possible that other human thrombin reagent constituents (e.g., solute concentration) somehow interact with the process of polymerization in an unknown fashion, accounting for the normal results obtained with the optical endpoint.

The normal and high FibC results following modest doses of cryoprecipitate suggest that a relatively small amount of normal fibrinogen in vivo is required to overcome the defective polymerization process. This provides weight to the concept of some sort of “threshold effect” over which the lateral protofibril stacking occurs sufficiently to produce a clot that is not translucent.

Jennings et al 8 reviewed the approach of multiple laboratories in the investigation of fibrinogen Longmont reference samples. They found discrepant results on different optical analyzers, with low FibC on ACL TOP and Sysmex CA660, and normal results on Sysmex CS2100, regardless of reagent. In accordance with other reports, they found a normal FibC result on the mechanical Stago STA-R Evolution. It is evident that the method of detection of fibrin clot formation can result in different FibC measurements. Mechanical detection methods reliably give normal FibC results; however, different optical analyzers and thrombin reagents can yield differing outcomes.

Acquired dysfibrinogenemia in the absence of liver disease is extremely rare. Our case emphasizes that even in older individuals, correlation with family results is warranted to ensure a congenital dysfibrinogenemia is not overlooked. This case highlights the frequent lack of a clear correlation between genotype and phenotype in congenital dysfibrinogenemias. In the laboratory assessment of unexplained cases of abnormal fibrinogen assays, a variety of clot detection methods and reagents may be required to detect fibrinogen Longmont.

## References

[1] Subcommittee on Factor XIII and Fibrinogen Casini A Undas A Palla R Thachil J de Moerloose P Diagnosis and classification of congenital fibrinogen disorders: communication from the SSC of the ISTH J Thromb Haemost 2018 16 09 1887 1890 30076675 10.1111/jth.14216

[2] Casini A Neerman-Arbez M Ariëns R A de Moerloose P Dysfibrinogenemia: from molecular anomalies to clinical manifestations and management J Thromb Haemost 2015 13 06 909 919 25816717 10.1111/jth.12916

[3] Hill M Dolan G Diagnosis, clinical features and molecular assessment of the dysfibrinogenaemias Haemophilia 2008 14 05 889 897 18564189 10.1111/j.1365-2516.2008.01795.x

[4] Lefkowitz J B DeBoom T Weller A Clarke S Lavrinets D Fibrinogen Longmont: a dysfibrinogenemia that causes prolonged clot-based test results only when using an optical detection method Am J Hematol 2000 63 03 149 155 10679806 10.1002/(sici)1096-8652(200003)63:3<149::aid-ajh8>3.0.co;2-#

[5] Lounes K C Lefkowitz J B Coates A I Hantgan R R Henschen-Edman A Lord S T Fibrinogen Longmont. A heterozygous abnormal fibrinogen with B beta Arg-166 to Cys substitution associated with defective fibrin polymerization Ann N Y Acad Sci 2001 936 129 132 11460470

[6] Lounes K C Lefkowitz J B Henschen-Edman A H Coates A I Hantgan R R Lord S T The impaired polymerization of fibrinogen Longmont (Bbeta166Arg–>Cys) is not improved by removal of disulfide-linked dimers from a mixture of dimers and cysteine-linked monomers Blood 2001 98 03 661 666 11468164 10.1182/blood.v98.3.661

[7] Grimley C Hill M Westby J Dolan G Appearances can be deceptive: a family with dysfibrinogenaemia, Fibrinogen Longmont that causes discrepant clotting times depending on the clot detection mechanism used, but appear not to bleed[abstract] Haemophilia 2006;12(26):

[8] Jennings I Kitchen S Menegatti M Potential misdiagnosis of dysfibrinogenaemia: data from multicentre studies amongst UK NEQAS and PRO-RBDD project laboratories Int J Lab Hematol 2017 39 06 653 662 28766854 10.1111/ijlh.12721

